# 16S rRNA Next-Generation Sequencing May Not Be Useful for Examining Suspected Cases of Spontaneous Bacterial Peritonitis

**DOI:** 10.3390/medicina60020289

**Published:** 2024-02-08

**Authors:** Chan Jin Yang, Ju Sun Song, Jeong-Ju Yoo, Keun Woo Park, Jina Yun, Sang Gyune Kim, Young Seok Kim

**Affiliations:** 1 Department of Internal Medicine, Soonchunhyang University Bucheon Hospital, Bucheon 14584, Republic of Korea; cjyang7@naver.com (C.J.Y.); 19983233@schmc.ac.kr (J.Y.); mcnulty@schmc.ac.kr (S.G.K.); liverkys@schmc.ac.kr (Y.S.K.); 2 GC Genome, Department of Laboratory Medicine, Green Cross Laboratories, Youngin 16924, Republic of Korea; sjusun277@gmail.com; 3 Preclinical Stroke Modeling Laboratory Weill Cornell Medicine, Burke Medical Research Institute, White Plains, NY 10605, USA; ballpark1114@gmail.com

**Keywords:** 16S rRNA next-generation sequencing, ascites, microbiome, spontaneous bacterial peritonitis

## Abstract

*Background and Objectives*: Ascites, often associated with liver cirrhosis, poses diagnostic challenges, particularly in detecting bacterial infections. Traditional methods have limitations, prompting the exploration of advanced techniques such as 16S rDNA next-generation sequencing (NGS) for improved diagnostics in such low-biomass fluids. The aim of this study was to investigate whether the NGS method enhances detection sensitivity compared to a conventional ascites culture. Additionally, we aimed to explore the presence of a microbiome in the abdominal cavity and determine whether it has a sterile condition. *Materials and Methods*: Ten patients with clinically suspected spontaneous bacterial peritonitis (SBP) were included in this study. A traditional ascites culture was performed, and all ascites samples were subjected to 16S ribosomal RNA gene amplification and sequencing. 16S rRNA gene sequencing results were interpreted by comparing them to positive and negative controls for each sample. *Results*: Differential centrifugation was applied to all ascites samples, resulting in very small or no bacterial pellets being harvested. The examination of the 16S amplicon sequencing libraries indicated that the target amplicon products were either minimally visible or exhibited lower intensity than their corresponding negative controls. Contaminants present in the reagents were also identified in the ascites samples. Sequence analysis of the 16S rRNA gene of all samples showed microbial compositions that were akin to those found in the negative controls, without any bacteria isolated that were unique to the samples. *Conclusions*: The peritoneal cavity and ascites exhibit low bacterial biomass even in the presence of SBP, resulting in a very low positivity rate in 16S rRNA gene sequencing. Hence, the 16S RNA sequencing method does little to enhance the rate of positive samples compared to traditional culture methods, including in SBP cases.

## 1. Introduction

Ascites is characterized as an abnormal accumulation of fluid in the abdominal cavity, with its primary causes being liver cirrhosis (60%), malignant tumors (26%), and tuberculous peritonitis (7%) [1]. The abdominal cavity, including the gut, serves as a frequent site for bacterial translocation, highlighting the importance of the microbiome in this context [2]. The microbiome’s composition is a critical factor in health and disease, influencing the development and progression of conditions such as ascites.

In conditions such as spontaneous bacterial peritonitis (SBP), where intra-abdominal bacterial infection is prevalent, especially among liver cirrhosis patients, bacteria penetrate the mesenteric lymph nodes and cause inflammation in the ascitic fluid due to decreased intestinal permeability [3]. SBP occurs in 10 to 30% of cirrhosis patients and can lead to severe complications such as hepatic encephalopathy, renal dysfunction, and potentially fatal sepsis if not diagnosed and promptly treated [4,5]. The limited sensitivity of traditional ascites culture methods, at about 40–50%, often results in negative outcomes in clinical practice [6]. This raises questions regarding the sensitivity of conventional culture tests and the actual bacterial presence in the abdominal cavity.

Meanwhile, next-generation sequencing (NGS) has shown promise as a more precise and sensitive method. Its application across diverse sample types underscores its superiority over traditional methods. For example, in respiratory infections, NGS has been reported to have a sensitivity as high as 90%, far surpassing conventional culture techniques [7,8]. Elsewhere, in urinary tract infections, NGS not only identified a wider array of pathogens but also provided more comprehensive resistance profiles than standard urine cultures [9,10]. This is particularly crucial in cases involving multidrug-resistant organisms where accurate pathogen identification can significantly impact treatment decisions.

Additionally, in low-biomass samples, such as cerebrospinal fluid, NGS has demonstrated remarkable accuracy in identifying causative organisms, even in instances where standard methods yielded no results [11,12]. This capability is particularly valuable in diagnosing infections in immunocompromised patients, where timely and accurate detection of pathogens can be life-saving. Furthermore, in gastrointestinal disorders, NGS has been effective in characterizing the gut microbiome, revealing complex bacterial communities that traditional cultures could not detect [13,14]. This has implications for understanding diseases such as inflammatory bowel disease and irritable bowel syndrome, where the gut microbiota plays a significant role.

Against that background, in this study, we aimed to assess NGS’s effectiveness when using 16S rRNA to enhance the sensitivity of the detection of bacteria in ascites fluid compared to traditional methods. Given NGS’s proven accuracy in other sample types, this study focused on whether NGS could improve the identification of bacteria in suspected cases of SBP, thereby addressing a significant diagnostic challenge.

## 2. Materials and Methods

### 2.1. Patients

From January to June 2022, among patients diagnosed with ascites due to portal hypertension or malignant ascites, clinically suspected cases of bacterial peritonitis were included in this study. Clinically suspected cases of bacterial peritonitis were defined based on clinical symptoms rather than diagnostic tests, defined as (i) mild-to-moderate abdominal pain or (ii) abdominal pain accompanied by fever. Inclusion criteria were as follows: (i) patients at least 19 years old, and (ii) hospitalized patients with cirrhosis. Pregnancy or identified secondary peritonitis were exclusion criteria. Informed consent was obtained from all patients who participated in this study. The samples were obtained via paracentesis, and ascites NGS tests were performed. In addition, we tested the ascites fluid cell count and ascites chemistry and we carried out a traditional ascites culture. The latter was performed using a standard blood culture consisting of an aerobic and anaerobic bottle set, with blood collected using an aseptic technique. The study protocol was approved by the Institutional Review Board of Soonchunhyang University Bucheon Hospital (IRB number SCHBC 2021-06-019-001) and followed the ethical guidelines of the World Medical Association’s Declaration of Helsinki. Written consent was obtained from all patients who participated in this study.

### 2.2. DNA Extraction

Collected ascitic fluids were transported under refrigeration to the genetic lab and the extraction process was started within 24 h. Most ascites samples were collected in quantities of around 500 mL. From each of these samples, 25 mL was randomly collected and tested. Sequencing was performed once per patient. We used different DNA extraction protocols for malignant ascites and cirrhotic ascites. Malignant ascites appears with a high proportion of malignant cells in the ascites, and if the proportion of mammalian cells is too high, it may interfere with the PCR of bacterial 16S rRNA. Therefore, after first removing the malignant cells, a two-step differential centrifugation was performed to concentrate the bacterial pellet. On the other hand, ascites caused by cirrhosis was judged to not have as high a proportion of malignant cells as malignant ascites, so only the first step, centrifugation, was performed.

For malignant ascites, initial centrifugation of 25 mL samples at 2770 rpm for 10 min was performed to separate mammalian cells. Then, the supernatants were removed and subsequent differential centrifugation was performed at 4000 rpm for 10 min. A 300 µL specimen including bacterial pellets at the bottom of the tube was harvested and processed for DNA extraction using a MagMAX™ Microbiome Ultra Nucleic Acid Isolation Kit (ThermoFisher Scientific, Waltham, MA, USA), according to the manufacturer’s instructions. Negative controls were simultaneously processed to check for the contamination of the DNA extraction kit.

For cirrhotic ascites, initial centrifugation with 25 mL samples at 4000 rpm was performed for 30 min, and then 1 mL from the bottom of the tube containing bacterial pellets was centrifuged at a higher speed of 14,000× *g* for 10 min to collect more bacterial cells. Ultimately, 300 µL of specimen including bacterial pellets was harvested and processed in the same way as described above. The negative controls for each DNA extraction batch were also simultaneously processed. To minimize the error in the experimental procedure, a positive control was also processed and analyzed, composed of six standard strains (*Pseudomonas aeruginosa*, *Escherichia coli*, *Stenotrophomonas maltophilia*, *Staphylococcus aureus*, *Streptococcus pneumoniae*, and *Enterococcus faecalis*). Each was cultured in the microbiological laboratory and 1 colony-forming unit was added to 6 mL phosphate-buffered saline (PBS); then, 250 µL of these strains combined was mixed with 25 mL of ascites sample 1 to form the positive control. The expected bacteria were detected well in the positive control, which proved that if bacteria were not detected in the ascites samples, it was not due to an error in the extraction process.

### 2.3. 16S rRNA Sequencing

In this study, we amplified and sequenced the V4 region of the 16S rRNA gene. The extracted DNA was then utilized to construct a 16S rRNA gene library, employing the NEXTflex 16S V4 Amplicon-Seq kit from BioO Scientific (Austin, TX, USA). The quality of the prepared library was assessed using the 4200 Tape Station System from Agilent Technologies (Santa Clara, CA, USA). Meanwhile, we conducted paired-end sequencing using the MiSeq Reagent Kit v2 nano on a MiSeq 2000 system, following the instructions provided by Illumina (San Diego, CA, USA). To evaluate the overall quality of the Illumina MiSeq paired-end sequencing (PE, 2 × 250 nucleotides), we incorporated 12% PhiX DNA from Illumina (USA) into the sequencing runs.

### 2.4. Bioinformatics Analysis

Primer trimming of the paired-end data was conducted using Cutadapt, and the resulting fastq files were imported into a QIIME2 data artifact, designated with a .qza extension. The DADA2 software v1.16 (Bioconductor) was employed for quality trimming, merging of paired-end sequences, and removal of chimeric sequences. The denoising parameters were set as follows: the length for forward reads was fixed at 200 bases, and for reverse reads, it was set at 160 bases. In the output table, features that had fewer than 10 reads or were present in only a single sample were eliminated. For taxonomic classification, filtered features were grouped and uniformly classified using the naïve Bayesian classifier, which was trained with the RefSeq reference databases. The q2-feature classifier, implemented in QIIME2, was utilized as the naïve Bayesian classifier without any modifications. The RefSeq reference databases served as the training dataset.

## 3. Results

### 3.1. Baseline Characteristics

Table 1 shows the baseline characteristics of the enrolled patients. This study included a total of 10 patients with clinically suspected peritonitis, composed of 3 patients with malignant ascites and 7 patients with ascites due to portal hypertension. The gender balance was 60% male and the mean age was 67.9 ± 10.5 years. Only two patients had a turbidity result of cloudy ascites, while the others were clear. The cell count results showed 42/µL to 1602/µL for the ascites white blood cells (WBCs) and that the percentage of polymorphonuclear leukocyte (PMN) ranged from 1% to 100%. We found results of 1.3 g/dL or higher for the serum ascites albumin gradient (SAAG) in patients with portal hypertension ascites, and in the patients with malignant ascites, two had a SAAG of less than 1.3 g/dL, while it was over 1.3 g/dL for one patient. Although the WBC of ascites increased, no bacteria were detected in the traditional culture.

### 3.2. Results of Ascites 16S rRNA Gene Sequencing Test

**Figure 1:**
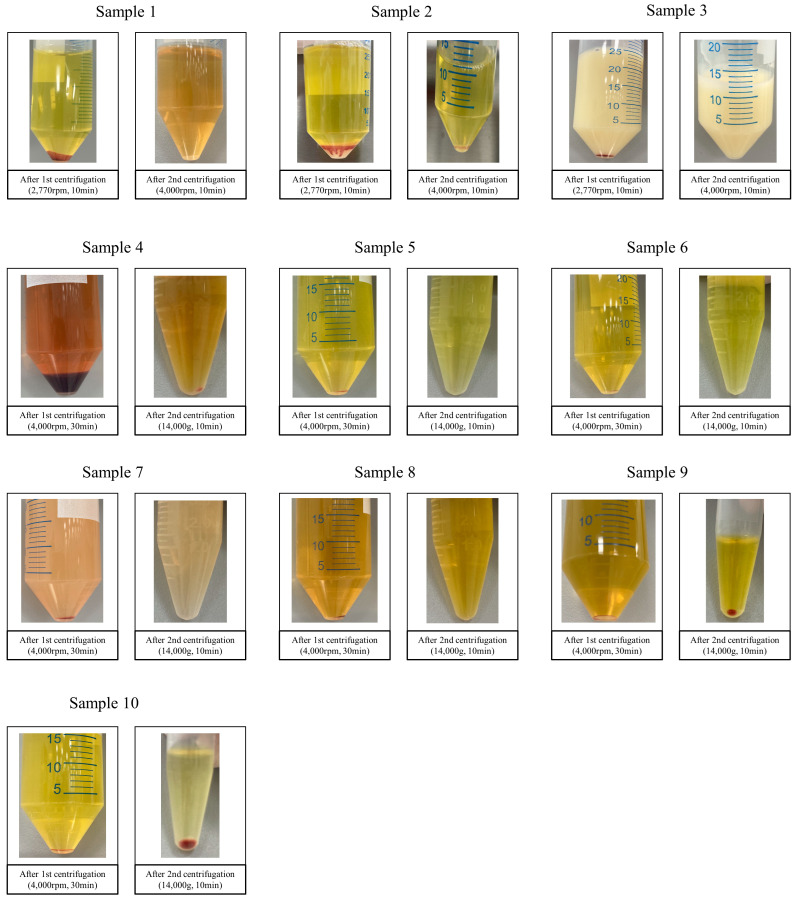
Specimens’ appearances following centrifugation under the given conditions.

**Figure 2:**
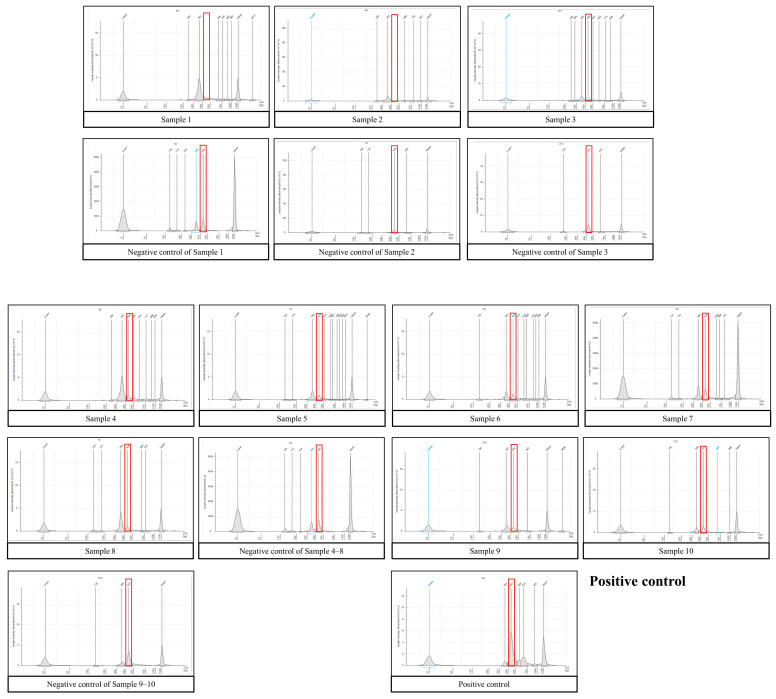
16S library for TapeStation analysis. The expected size of the target amplicon is approximately 450 bases. The red box indicates the expected target region. The target amplicon products in samples are almost invisible or smaller than those of the negative control, indicating that bacteria were not present or barely present in ascites compared to the negative control. Note that the scale of the Y-axis varies between samples.

**Figure 3:**
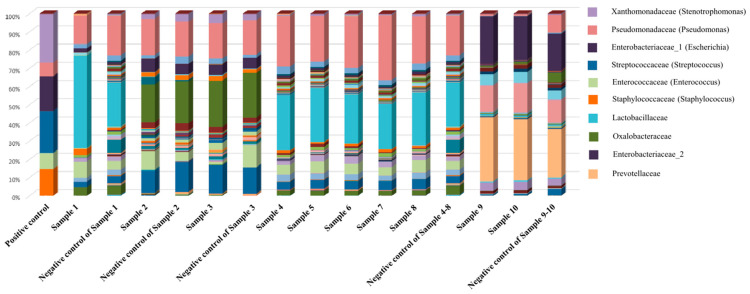
Sequencing results including the negative and positive controls. The results are similar to those of the negative control, indicating that no bacteria were detected in the specimens (including the bacterial pellets), while the results for the positive control showed the compositions of the six standard strains.

First, centrifugation was conducted, a common laboratory practice used to harvest bacteria from clinical specimens [15]. After differential centrifugation was applied to the ten ascites samples, small pellets were harvested from two malignant samples (Patients No. 1 and 2), while no pellets were visible in the rest of the samples (Figure 1). In this test, the pellet that settles after centrifugation is concentrated bacteria or mammalian cells. If the pellet is small or barely visible, this means there are very few bacteria in the sample, in this case, suggesting that the ascites has a very low biomass of bacteria. Next, prepared 16S amplicon sequencing libraries were analyzed using a bioanalyzer. The target amplicon products in the samples were almost invisible or lower than for the negative control (Figure 2), indicating that bacteria were not present or were barely present in the ascites samples compared to the negative control. The target amplicon refers to the amplification product of bacteria in the sample. Since contaminated microbial nucleic acids existed in the reagent, target amplicon products were created even in negative controls that did not contain samples. The fact that the target amplicon products of the ascites samples were smaller or less visible compared to the negative control showed that there was barely any formation of the target amplicon by sample-specific bacteria other than amplification of contaminated microbial nucleic acids in the reagent, i.e., the microbial sequences amplified from the ascites samples were reagent-derived rather than sample-specific. Finally, 16S rRNA gene sequencing was performed and analyzed. The sequencing results for the samples showed that their microbial compositions were similar to that of the negative control, and any sample-specific bacteria were isolated (Figure 3). Meanwhile, the microbial composition of the positive control mixture with six standard strains revealed that all experimental procedures appeared to be appropriate. Furthermore, through comparison with the positive control, we confirmed that 16S amplicon sequencing did not detect bacteria in the ascites samples in this study. That is to say, in the positive control, we detected bacterial sequences that were not detected in the negative control, which showed that bacteria existed specifically in the positive control, and since bacterial sequences that were not detected in the negative control were also not detected in the ascites sample, it can be said that ascites sample-specific bacteria were not detected.

### 3.3. Clinical Course of the Patients

The ten patients who participated in this study were clinically diagnosed with bacterial peritonitis, and empirical antibiotics such as third-generation cephalosporins were administered immediately after paracentesis regardless of the 16S rRNA gene sequencing results. After the administration of these antibiotics, symptoms such as abdominal pain and fever improved in all patients on average on the third day of administration (range 2–7 days).

## 4. Discussion

The results of our study indicate that while 16S rRNA gene sequencing offers high sensitivity, its effectiveness in ascites samples is limited due to low bacterial biomasses and potential contamination. This raises concerns about the suitability of 16S rRNA gene sequencing for diagnosing bacterial infections in ascites, emphasizing the need for improved techniques and protocols in such low-biomass samples.

Compared to a conventional culture, 16S rRNA gene sequencing can detect causative bacteria in a smaller biomass thanks to its high sensitivity [16]. Furthermore, recent advances in technologies are greatly improving the testing speed, and there has been increasing attention on research aimed at developing a next-generation sequencing method with which to identify the causative bacteria of infection [17,18]. The conventional culture method using a blood culture bottle to identify the causative bacteria for ascites has only 40 to 50% sensitivity [19]. Therefore, attempts have been made to identify the causative bacteria in patients suspected of having SBP by utilizing the advantages of 16S rRNA gene sequencing [20,21,22]. However, our study found that despite the high sensitivity of 16S rRNA gene sequencing, it did not demonstrate an advantage over conventional culture methods in detecting bacteria in ascites samples, including in cases of suspected SBP. We attribute this lack of success to the low biomass in ascites fluid, which makes bacterial detection via 16S rRNA gene sequencing challenging since it significantly impacts the method’s sensitivity. In low-biomass samples such as those in ascites, the reduced presence of bacterial DNA makes it difficult for 16S rRNA gene sequencing to detect and accurately identify bacterial species. This creates challenges in distinguishing between true microbial signals and background noise or contaminants, which are more pronounced in low-biomass conditions. Consequently, the effectiveness of 16S rRNA gene sequencing in such scenarios is limited, raising concerns about its reliability for diagnosing bacterial infections in ascites, including in suspected SBP cases.

Additionally, the presence of contaminants in the 16S rRNA gene sequencing process further complicates the interpretation of results. Although the advantage of 16S rRNA gene sequencing is that it can be used to detect the causative bacteria with only a small amount of biomass, increasing the sensitivity when using a DNA extraction kit, adopting this approach is considered to introduce a risk of contamination compared to the existing culture method, as was observed in our results. These factors combined suggest that while 16S rRNA gene sequencing has the potential for high accuracy, its application in low-biomass samples such as ascites fluid needs careful consideration and refinement.

Several studies including ours have been conducted to detect the causative bacteria via 16S rRNA gene sequencing in patients suspected of ascitic fluid infection. In some studies, this sequencing approach had significantly higher sensitivity for detecting the causative bacteria when compared to conventional culture methods, and it also confirmed the presence of anaerobic bacteria and polymicrobial infections that were difficult to identify with conventional culture methods [23,24]. Fast detection of the causative bacteria via 16S rRNA gene sequencing in patients with ascitic fluid infections led to the implementation of appropriate targeted antimicrobial therapy and a reduction in mortality [25]. However, to our knowledge, no studies have addressed the possibility that 16S rRNA gene sequencing results in ascites are caused by contamination. As well as beginning to fill this research gap, in this study, we also considered the possibility that a negative result might have derived from inappropriate sample processing. However, in the laboratory where this study was conducted, urine samples (low biomass) are also tested by applying a similar centrifugation pretreatment method with the same extraction protocol, and the positive rate in urine samples is 70–80% [9,10]. Therefore, we do not believe the pretreatment protocol applied to the ascites samples was inappropriate. Furthermore, we applied a different centrifugation method to see if different results were obtained. Sample 2 was tested using the density-gradient centrifugation method using a 10 m Percoll, and the result we obtained was similar.

To date, microbiome detection protocols in ascites fluid exist in various forms and have not been clearly established. Ascites involves low biomass, and in the present study, we used a 16S rRNA gene sequencing protocol with a positivity rate of more than 70% in urine (which has one of the lowest biomasses), but the positivity rate in ascites was very low [9,10]. This suggests that the microbial burden of ascites is much lower than that in urine samples with cystitis or almost absent. We also performed the 16S rRNA gene sequencing test in patients with suspected SBP, but the result was negative. These results indicated that typical cirrhotic ascites samples without infection are sterile. There are microbial floras in the gastrointestinal tract, skin, respiratory tract, and genital organs which make contact with the outside world, but the abdominal cavity is internal, so the flora is considered to be much lower than in other parts of the body or even absent. Usually, an imbalance of flora in a specific area, called dysbiosis, is associated with various health issues, including inflammation [26]. However, it is difficult for the abdominal cavity and peritoneal tissues to enter into dysbiosis since they have little flora. Instead, there is a high possibility that the infection brought about with the inoculation of bacteria to form a sterile space occurs as a result of the translocation of bacteria in the intestine to the abdominal cavity [27,28,29].

The sterile condition of the abdominal cavity has already been confirmed through other 16S rRNA gene sequencing studies. Even metagenomic sequencing, which has a higher detection rate than 16s RNA, showed that 24% of patients with suspected SBP were negative [30]. In the present study, bacterial peritonitis, caused by bacterial infection in the abdominal fluid, induced the majority of ascites, but the concentration of bacteria after collection was relatively low, reflecting that it may be difficult to detect via centrifugation. In addition, the sample collection for ascites was conducted after phagocytosis of bacteria by leukocytes, which decreased the positivity rate of 16S rRNA gene sequencing [21].

Microbes are the predominant life form on Earth and extracellular DNA is found in all ecosystems [31]. Accordingly, bacterial DNA contamination can occur in laboratories as well as in experimental reagents, including DNA extraction kits and other reagents of molecular biology grade, consequently affecting sequencing datasets [32]. A previous study found that reagent contamination mainly originated from a DNA extraction kit, with background contaminants in the DNA extraction kit calculated as approximately 500 copies per μL of elution volume (though the amount varied depending on the type of kit) [33]. Therefore, in the case of a low-biomass sample, if there is a smaller amount of bacterial DNA than contaminant-originated DNA in the reagent, it is difficult to uncover the presence of bacteria in the sample. On the topic of 16S rRNA gene sequencing contamination, it is hard to accurately compare the results from previous studies as they did not describe their certainty of reagent contamination in the same way that we have in our study. However, the possibility of contamination of PCI reagents has been reported in several articles [34,35,36,37]. One 16S rRNA gene sequencing study using ascites reported that 24% of patients with suspected SBP had polymicrobial infection [30]. Considering that the possibility of polymicrobial infection is not high in patients with SBP, unless a patient has secondary peritonitis, then there is a significant possibility of contamination. Failure to use appropriate antibiotics, if they are administered for infection pathogens rather than contaminants, may increase mortality; in addition, it may unnecessarily raise the medical resources consumed if one course of antibiotics must later be followed by a different course. Similarly, when interpreting the results of traditional microbial cultures, it is important to rule out the possibility of contamination in order to determine the origins of the cultured bacteria, i.e., whether they are actual infection pathogens. Therefore, it is meaningful that our study suggests the possibility of contamination in 16S rRNA gene sequencing. In particular, in samples with a low microbial burden, such as in ascites, contaminants from the DNA extraction kit can be detected more significantly, so it may not be appropriate to test ascites samples with 16S rRNA gene sequencing [38].

When interpreting our findings, note that our study’s limitations include its small sample size and the lack of validation with a separate sample set. These factors may affect the generalizability and robustness of the findings. The small sample size limited the statistical power to detect differences and means the findings may not represent the broader patient population. Additionally, the lack of validation with another sample set means the results might not be replicable in different settings or populations.

Future research, in addition to overcoming this study’s limitations, could explore more sensitive methods such as whole-genome sequencing, which might provide better detection in low-biomass samples such as those in ascites [39]. Further studies should also assess the clinical utility and cost-effectiveness of 16S rRNA gene sequencing in this context. Beyond that, investigating improved sample preparation techniques and contamination control methods could enhance the accuracy of 16S rRNA gene sequencing in diagnosing bacterial infections in ascites, including in cases of suspected SBP. Research in these directions could significantly contribute to optimizing diagnostic approaches in medical practice.

The key conclusion of this study is that using 16S rRNA gene sequencing in ascites samples faces significant challenges and limitations. These include the low biomass in ascites, which complicates the detection of bacteria, and the presence of contaminants that can skew 16S rRNA gene sequencing results. These issues highlight the need for improved methodologies and caution in interpreting 16S rRNA gene sequencing data from ascites samples, including in clinical diagnostics for conditions such as SBP.

## Figures and Tables

**Table 1:** Baseline characteristics of the patients.

No.	Sex	Age (Years)	Etiology	Ascites Culture	Ascites Color	Ascites Turbidity	Ascites WBC (Cells/µL)	Ascites PMN (%)	SAAG (g/dL)	Ascites Protein (mg/dL)
1	F	78	Ovarian cancer	Absence of bacterial growth	Yellow	Clear	135	72	0.9	1270
2	F	69	Ovarian cancer	Absence of bacterial growth	Yellow	Cloudy	1602	16	0.4	5291
3	F	64	Colon cancer with liver metastasis	Absence of bacterial growth	Yellow	Clear	198	6	1.7	775
4	M	59	HCV-LC	Absence of bacterial growth	Red	Cloudy	154	100	2.1	1027
5	F	89	Biliary cirrhosis	Absence of bacterial growth	Yellow	Clear	210	65	1.7	1254
6	M	62	HBV-LC	Absence of bacterial growth	Yellow	Clear	77	49	2.1	1151
7	M	64	NBNC-LC	Absence of bacterial growth	Dark yellow	Cloudy	108	4	1.8	1163
8	M	63	NBNC-LC	Absence of bacterial growth	Yellow	Clear	42	2	2.3	568
9	M	52	NBNC-LC	Absence of bacterial growth	Yellow	Clear	42	1	2.7	892
10	M	79	NBNC-LC	Absence of bacterial growth	Yellow	Clear	144	26	1.7	775

Abbreviations: WBC, white blood cell; PMN, polymorphonuclear neutrophil; SAAG, serum ascites albumin gradient; F, female; M, male; HCV, hepatitis C virus; LC, liver cirrhosis, HBV, hepatitis B virus; NBNC, non-HBV/non-HCV.

## Data Availability

The datasets generated and/or analyzed during the current study are available from the corresponding author on reasonable request.
